# The Prevalence of and Factors Associated With Anxiety and Depression Among Working-Age Adults in Mainland China at the Early Remission Stage of the Coronavirus 2019 Pandemic

**DOI:** 10.3389/fpsyg.2022.839852

**Published:** 2022-03-31

**Authors:** Haixia Xie, Xiaowei Huang, Qi Zhang, Yan Wei, Xuheng Zeng, Fengshui Chang, Shuyin Wu

**Affiliations:** ^1^Department of Social Work, Shanghai Yangzhi Rehabilitation Hospital (Shanghai Sunshine Rehabilitation Center), School of Medicine, Tongji University, Shanghai, China; ^2^Department of Gastroenterology and Hepatology, Tongji Hospital Affiliated to Tongji Medical College of Huazhong University of Science and Technology, Wuhan, China; ^3^School of Community and Environmental Health, Old Dominion University, Norfolk, VA, United States; ^4^China Research Center on Disability, School of Public Health, Fudan University, Shanghai, China; ^5^School of Health Management, Bengbu Medical College, Bengbu, China

**Keywords:** COVID-19, depression, anxiety, working-age population, adult, Mainland China

## Abstract

**Background:**

The Coronavirus 2019 (COVID-19) outbreak has led to a considerable proportion of adverse psychological symptoms in different subpopulations. This study aimed to investigate the status of anxiety and depression and their associated factors in the adult, working-age population in Mainland China at the early remission stage of the COVID-19 pandemic.

**Methods:**

An online study was conducted among 1,863 participants in 29 provinces in Mainland China from March 23 to 31, 2020. Their mental health was evaluated by the generalized anxiety disorder scale (GAD-7) and the patient health questionnaire (PHQ-9). Descriptive analysis, Chi-square, and multiple logistic regressions were applied.

**Results:**

About 44.5% of the participants had anxiety, 49.2% had depression, and 37.9% showed a combination of depression and anxiety. Around 83.7% of the participants claimed that the pandemic had a negative impact on their medical needs, which was the primary predictor of mental health, the degree of impact being positively related to the prevalence of anxiety and depression. More chronic diseases, moderate to bad self-rated health, severe perceived infection risk, and younger age group were the common risk factors for anxiety and depression. Having no children, unemployment, and a college-level educational background were associated with higher anxiety prevalence, whereas unmarried participants were correlated with higher depression prevalence.

**Conclusion:**

The working-age population showed a relatively high risk of anxiety and depression in Mainland China at the early remission stage of the pandemic. To improve medical services capacity for routine and delayed medical service needs should be a part of policy-makers’ priority agenda during this period of crisis.

## Introduction

The first four coronavirus 2019 (COVID-19) cases were reported in Wuhan in December 2019 (Gong et al., 2020), and the outbreak was declared a Public Health Emergency of International Concern by the World Health Organization (WHO) on January 31, 2020 (Department of International Cooperation, 2020). The WHO made the assessment that COVID-19 could be characterized as a pandemic on March 11, 2020 (World health organization, 2020). As of March 10, 2022, the pandemic has resulted in 112,940 confirmed cases and 4,636 deaths in Mainland China (National Health Commission of the PRC, 2022). Worldwide, there have been over 450 million confirmed cases of COVID-19, including approximately 6.02 million deaths, reported to the WHO by March 10, 2022 (World health organization, 2022). To curb this unprecedented public health crisis, at the initial stage of the pandemic the Chinese government launched the most comprehensive, stringent, and thorough prevention and control campaign to prevent it from spreading, i.e., Wuhan was locked down starting at 10:00 a.m. on January 23, 2020 (Liu et al., 2020a, 2021). People living in Mainland China were forced to isolate themselves in their homes, to wear face masks, and to practice social distancing in public places, and all communities were requested to strengthen the management of population movement (including issuing entry permits) and to monitor visitors’ temperature.

Quarantine/isolation and other prevention methods are effective ways to control virus transmission but may have negative effects on psychological health (Henssler et al., 2021). In the face of this public health crisis, all the subpopulations (e.g., healthcare providers, college students, teenagers, the general public, etc.) have been experiencing anxiety, depression, insomnia, and acute stress (Wu et al., 2021b; Yu et al., 2021). Much research and many reviews have been conducted globally to explore the mental health of different population groups (Akintunde et al., 2021; Wu et al., 2021b). Wang et al. showed that 51.6 and 59.2% of Chinese participants (≥15 years) at the remission stage of the COVID-19 epidemic reported having symptoms of anxiety and depression, respectively (Wang et al., 2021f). Another study reported that the rates of mental health symptoms among the adult population (≥18 years) in China were 27.9% for depression and 31.6% for anxiety (Shi et al., 2020). Risk factors for anxiety and depression may have been lower level of education, female gender, younger age, healthcare workers, lower-income, place of residence, poor self-rated health, history of chronic diseases, not being married, isolation/quarantine experience, difficulty accessing medical aids, and having relatives or acquaintances infected with COVID-19 (Cao et al., 2020; Duan et al., 2020; Peng et al., 2020; Ren et al., 2020; Talevi et al., 2020; Gong et al., 2021; Guo et al., 2021; Zhu et al., 2021). However, there existed disparities for some factors (e.g., education and quarantine) among different studies of different quality and using different tools to study the same mental health outcomes (Luo et al., 2020; Wang et al., 2021e). With reference to the Bulletin of China Seventh Census carried out in 2020, the population aged 15–59 constitutes 63.35% (894.38 million) of the citizens in Mainland China (National Bureau of Statistics, 2021). This group might be more adversely impacted than other groups due to the disruption of their normal jobs and lives, thus, this group should be focused upon for support and efforts to ameliorate their situation (Ahmed et al., 2020; Wang et al., 2020c). However, the mental effects of COVID-19 for the adult, working-age population (age ≥ 18 years, and men: age ≤ 59 years, women: age ≤ 54 years) have not yet been studied in China. This study aimed to evaluate the status of anxiety and depression and their associated factors among the adult, working-age population in Mainland China and provide a reference for strengthening social support and psychological assistance measures for this group at the early remission stage of the pandemic.

## Materials and Methods

### Participants and Study Design

From the point of view of newly infected cases, the COVID-19 pandemic in Mainland China reached its peak in February 2020. This cross-sectional study was done from March 23 to 31, 2020. During this period, the COVID-19 pandemic in China was already controlled in the sense of having very limited newly infected cases (<100) being reported every day (National Health Commission of the PRC, 2020). The study inclusion criteria included (a) adult Chinese people (age ≥ 18 years, and men: age ≤ 59 years, women: age ≤ 54 years, given that legal retirement ages in Mainland China in 2020 were 60 for men and 55 for women) living on the mainland, (b) without reported disabilities, and (c) willing to participate in the survey. The exclusion criteria were participants who: (a) could not satisfy the inclusion criteria; (b) filled out the questionnaires within 100 s, (c) were full-time students, and (d) submitted the questionnaires with logical errors.

### Recruitment Process and Data Collection

The data were anonymously collected by self-response questionnaires. The electronic questionnaires were completed *via* the Wenjuanxin platform (a frequently used online survey tool). The investigators in Shanghai, Wuhan, and Bengbu were responsible for handing out online questionnaires in WeChat groups (a most commonly used social media platform in China) on their social network, including relatives, friends, classmates, and so on. A snowball sampling strategy was applied, and the responders were encouraged to recruit other people. The questionnaire could be submitted only after it was completed, and each mobile phone could only be used once in order to prevent duplicate submissions. An online written informed consent was obtained from the participants *via* the Wenjuanxin platform before the start of the survey. This study was approved by the ethics committee of the School of Public Health at Fudan University in Shanghai, China (IRB#2020-03-0813). About 2,253 participants submitted the questionnaire successfully. After quality control, 1,863 participants from 29 provinces (municipalities or autonomous regions) were included, with the four leading regions being Anhui, Hubei, Shanghai, and Hebei, which constituted 58.3% of our participants.

### Instruments

#### Sociodemographic Variables

Personal and family characteristics included the participants’ sex, age, and primary residence (specific to province and city) since mid-January, 2020, region type, educational background, marital status, employment, monthly personal income, and number of children. The region type was described as either urban or rural. Marital status was classified as unmarried, married, divorced, or widowed. Employment status was classified as unemployed and employed. The state of work resumption during the data collecting period was classified into not at work, at work, or in quarantine (home quarantine or centralized quarantine).

#### Chronic Diseases and Self-Rated Health

The kinds of chronic diseases were calculated based upon the following multiple choice question: Do you have chronic diseases that have been diagnosed by the doctor in the last 6 months? (a) hypertension, (b) diabetes, (c) gastroenteritis, (d) rheumatoid arthritis, (e) cerebrovascular disease, (f) intervertebral disc disease, (g) chronic obstructive pulmonary disease, (h) ischemic heart disease, (i) gallstones and cholecystitis, (j) peptic ulcer, (k) prostatic hyperplasia, (l) cataract, (m) asthma, (n) chronic pharyngolaryngitis, (o) tumor, (p) chronic hepatitis, and (q) other (please fill in the blank)__________. Self-rated health was evaluated by a five-level Likert measurement: very dissatisfied, dissatisfied, neither satisfied nor dissatisfied, satisfied, and very satisfied.

#### Level of Anxiety/Depression

Anxiety/depression was measured using the seven-item generalized anxiety disorder scale (GAD-7; Spitzer et al., 2006) and the nine-item patient health questionnaire (PHQ-9; Kroenke et al., 2001), which inquired about symptoms of anxiety/depression during the past 2 weeks using a four-point scale: not at all, several days, more than half the days, and nearly every day. Each item of the scale ranges from 0 to 3 points, with a total possible score of 21/27 points. A higher score suggests a higher level of anxiety/depression severity. The Chinese Version of the GAD-7 and PHQ-9 have been validated in Mainland China (Wang, 2013). Scores of 5, 10, and 15 represent cut-off points for mild, moderate, and severe anxiety/depression, respectively (Wang, 2013). These two measures showed very high internal consistency reliability (both Cronbach’s alphas of 0.94) in our study.

#### Perceived Risk of COVID-19 Infection

The perceived risk of COVID-19 infection was investigated with multiple choice questions whose items included: (a) I and/or my family member was infected, (b) reported cases of infection among my relatives and/or good friends, (c) reported cases of infection in the corridor of my living unit and/or other corridors in my residential building, (d) reported cases of infection in the organization where I worked, (e) reported cases of infection in the neighbourhood/village that I lived, (f) reported cases of infection in the street/town that I lived, (g) reported cases of infection in the district/county that I lived, (h) none, and (i) unclear.

#### Impact on Medical Services Needs

The impact on the participant’s and their family’s medical services needs from the pandemic was measured with a five-item Likert scale: not at all, a little, a moderate amount, very much, and an extreme amount.

### Statistical Methods

In this study, the descriptive analysis included the frequency and percentages of the categorical variables, and the means of all scale variables with SDs. The prevalence of anxiety and depression was reported with a 95% CI. The individual perceived risk of COVID-19 infection was recoded in four categories: none, unclear, and moderate (reported infected cases in the street/town or district/county that I lived), and severe (at least one choice of the five conditions: (a) I and/or my family member was infected, (b) reported cases of infection among my relatives and/or good friends, (c) reported cases of infection in the corridor of my living unit and/or other corridors in my residential building, (d) reported cases of infection in the organization where I worked, and (e) reported cases of infection in the neighborhood/village that I lived). Self-rated health was recoded into three categories (bad: very dissatisfied, dissatisfied; moderate: neither satisfied nor dissatisfied; and good: satisfied and very satisfied). Chi-square or Fisher’s exact tests were performed in the univariate analysis of anxiety and depression. The Holm-Bonferroni adjustment was made for multiple comparisons in three or more groups of the categorical variables when the result of the chi-square tests indicated a statistical significance (Stephanie, 2021). Variables with significant differences were included in the multiple logistic regression models as independent variables, and the forward stepwise strategy was applied. All the independent variables were changed to dummy variables in the logistic regression analysis. The dependent variable was mild-to-severe anxiety/depression status (scored five or more). If the value of *p* of the normality test (Kolmogorov–Smirnov test) was ≤0.05, Spearman’s rho correlations were calculated in nonparametric analysis. All statistical tests were two-sided with a significant value of *p* < 0.05. All the analyses were run by the statistical package for social sciences for windows (SPSS for Windows 13.0, SPSS Inc., Chicago, IL, United States).

## Results

### Characteristics of the Participants

The personal and family characteristics of the participants are presented in Table 1. Among the 1,863 working-age participants, 319 (17.1%) persons were from Wuhan, the provincial capital of Hubei, which was the COVID-19 epicenter in Mainland China, and 3.4% were from Hubei (excluding Wuhan). The mean age (±SD) of the participants was 33.7 ± 8.7 years. Around 62.1% of the participants were at work during the data collecting period, 31.6% were not at work, and another (6.3%) were in quarantine.

**Table 1:** Personal and family characteristics of 1,863 participants.

Variables		*n*	%	Variables		*n*	%
Province/city	Wuhan	319	17.1	Region	Urban	1,602	86.0
	Hubei province^a^	64	3.4		Rural	261	14.0
	Other provinces	1,480	79.5	Employment	Unemployed	239	12.8
Sex	Male	934	50.1		Employed	1,624	87.2
	Female	929	49.9	Monthly personal income (RMB)	> 8,000	609	32.7
Marital status	Unmarried	494	26.5		6,001–8,000	388	20.8
	Married	1,314	70.5		4,001–6,000	426	22.9
	Divorced or widowed	55	3.0		≤ 4,000	440	23.6
Age (years)	18–29	612	32.9	Number of children	0	490	26.3
	30–39	783	42.0		≥1	1,373	73.7
	40–49	367	19.7	Status of work resumption	Not at work	588	31.6
	50–59	101	5.4		At work	1,157	62.1
Educational background	Junior high school or below	98	5.3		In quarantine	118	6.3
	Senior high school^b^	291	15.6	Kinds of chronic diseases	0	1,360	73.0
	Junior or regular college	1,109	59.5		1	308	16.5
	Graduate	365	19.6		≥2	195	10.5

a *Excluding Wuhan*.

b *Including secondary vocational school/technical school*.

### Perceived Risk of COVID-19 Infection

Around 30.4 and 32.4% of the participants reported moderate and severe perceived risk of COVID-19 infection, respectively (Table 2). The people in Hubei province reported higher perceived risk of COVID-19 infection than those of other provinces in Mainland China, and the highest percentage of high perceived risk of COVID-19 infection was observed in Wuhan, as expected.

**Table 2:** Participants’ perceived risk of Coronavirus 2019 (COVID-19) infection, by geographic area.

Province/city	None		Unclear		Moderate		Severe	
	*N*	%	*N*	%	*N*	%	*N*	%
Hubei province^a^	33	8.6	26	6.8	39	10.2	285	74.4
Wuhan^b^	23	7.2	19	6.0	18	5.6	259	81.2
Other cities in Hubei Province	10	15.6	7	10.9	21	32.8	26	40.6
Other provinces	584	39.5	50	3.4	528	35.7	318	21.5
Total	617	33.1	76	4.1	567	30.4	603	32.4

a *χ*^2^* = 424.4, *p* < 0.01 (Compared to other provinces)*.

b *χ*^2^* = 56.8, *p* < 0.01 (Compared to other cities in Hubei province)*.

### Overall Health of the Participants and General Impact on Medical Services Needs

Around 27.0% of the participants suffered chronic diseases. Of the 1,863 participants, 62.2% had good self-rated health, and 7.9/29.9% reported bad/moderate health, respectively. The majority of the participants (83.7%) claimed a negative impact on their medical services needs in the pandemic, and the percentage of those reporting a big impact (very much to an extreme amount) on medical services needs was 15.4%, while the percentages of those reporting a little and a moderate amount were 49.7 and 18.6%, respectively.

### Prevalence of Anxiety and Depression

**Figure 1:**
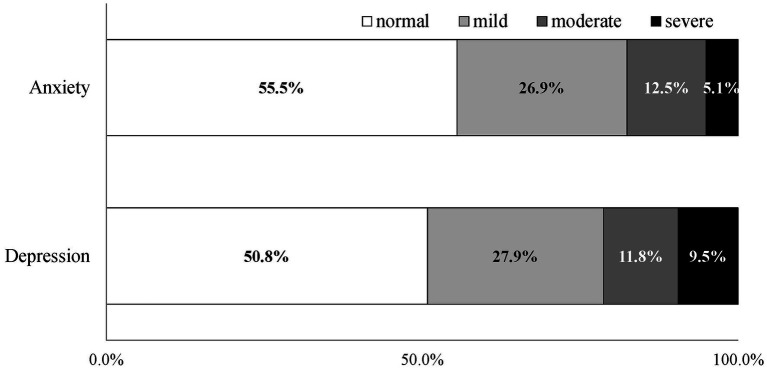
Stacked bar charts showing the severity of anxiety and depression of the adult, working-age population in Mainland China at the early remission stage of the COVID-19 pandemic.

The mean (SD) of the GAD-7 and PHQ-9 were 4.89 (4.93) and 5.87 (5.92), and the prevalence (95% CI) of possible anxiety and depression (Figure 1) were 44.5% (42.2%–46.8%) and 49.2% (46.9%–51.4%), respectively. Moderate and severe anxiety and depression could be observed in 17.6% (15.9%–19.3%) and 21.3% (19.4%–23.2%) of our participants, respectively. 37.9% (35.7%–40.1%) of the participants showed a combination of depression and anxiety (CDA), and the prevalence of moderate-to-severe CDA was 14.3% (12.7%–15.9%). The spearman ranking correlation coefficient of the GAD-7 and PHQ-9 scores was 0.81 (*p* < 0.01).

### Factors Associated With Anxiety and Depression

Univariate analysis (Table 3) revealed that the common possible associated factors of anxiety and depression were age, marital status, number of children, kinds of chronic diseases, individual perceived risk of COVID-19 infection, self-rated health and impact on medical service needs. Meanwhile, province/city, educational background, and employment status were the possible factors associated with anxiety. The participants in Wuhan reported a higher prevalence of possible anxiety than people in other regions (*χ^2^* = 10.39*, p* < 0.01).

**Table 3:** Univariate analysis of anxiety and depression of the adult, working-age population in Mainland China in the COVID-19 pandemic.

Variables		Anxiety (*N*, %)	*χ^2^*	*p*	Depressio*n* (*N*, %)	*χ^2^*	*p*
Province/city	Wuhan	168 (52.7)	10.40	0.01	158 (49.5)	4.64	0.10
	Hubei province^a^	27 (42.2)			23 (35.9)		
	Other provinces	634 (42.8)			735 (49.7)		
Region	Urban	712 (44.4)	0.01	0.91	785 (49.0)	0.13	0.72
	Rural	117 (44.8)			131 (50.2)		
Sex	Male	400 (42.8)	2.12	0.15	458 (49.0)	0.01	0.91
	Female	429 (46.2)			458 (49.3)		
Age (years)	18–29	302 (49.3)	16.62	<0.01	334 (54.6)	22.50	< 0.01
	30–39	352 (45.0)			390 (49.8)		
	40–49	142 (38.7)			158 (43.1)		
	50–59	33 (32.7)			34 (33.7)		
Educational background	Junior high school or below	39 (39.8)	9.27	0.03	44 (44.9)	4.12	0.25
	Senior high school^b^	121 (41.6)			133 (45.7)		
	Junior or regular college	525 (47.3)			566 (51.0)		
	Graduate	144 (39.5)			173 (47.4)		
Marital status	Unmarried	256 (51.8)	15.82	<0.01	278 (56.3)	15.77	< 0.01
	Married	546 (41.6)			607 (46.2)		
	Divorced or widowed	27 (49.1)			31 (56.4)		
Employment	Employed	704 (43.3)	6.76	0.01	787 (48.5)	2.54	0.11
	Unemployed	125 (52.3)			129 (54.0)		
Monthly personal income (RMB)	>8,000	259 (42.5)	1.84	0.61	290 (47.6)	4.50	0.21
	6,001–8,000	174 (44.8)			193 (49.7)		
	4,001–6,000	199 (46.7)			227 (53.3)		
	≤4,000	197 (44.8)			206 (46.8)		
Number of children	None	254 (51.8)	14.50	<0.01	272 (55.5)	10.70	<0.01
	≥1	575 (41.9)			644 (46.9)		
Status of work resumption	Not in work	269 (45.7)	1.98	0.37	301 (51.2)	2.03	0.36
	In work	502 (43.4)			554 (47.9)		
	In quarantine	58 (49.2)			61 (51.7)		
Kinds of chronic diseases	0	541 (39.8)	53.40	<0.01	598 (44.0)	69.96	<0.01
	1	161 (52.3)			177 (57.5)		
	≥2	127 (65.1)			141 (72.3)		
Individual perceived risk of COVID-19 infection	None	235 (38.1)	58.80	<0.01	265 (42.9)	30.90	<0.01
	Unclear	24 (31.6)			35 (46.1)		
	Moderate	226 (39.9)			265 (46.7)		
	Severe	344 (57.0)			351 (58.2)		
Self-rated health	Bad	87 (58.8)	61.63	<0.01	90 (60.8)	71.36	<0.01
	Moderate	308 (55.3)			345 (61.9)		
	Good	434 (37.5)			481 (41.5)		
Impact on medical services needs	None	81 (26.7)	110.49	<0.01	95 (31.4)	95.09	<0.01
	A little	395 (42.7)			431 (46.6)		
	Moderate	154 (44.4)			188 (54.2)		
	Big^c^	199 (69.1)			202 (70.1)		

a *Excluding Wuhan*.

b *Including secondary vocational school/technical school*.

c *Very much to an extreme amount*.

The multivariate logistic analysis (Table 4) found that the impact on medical service needs, kinds of chronic diseases, self-rated health, age, and individual perceived risk of COVID-19 infection were the common associated factors for anxiety and depression among the adult, working-age population. The percentages of possible anxiety and depression among the participants who reported an impact on their medical service needs were higher than those of participants who reported no impact: the more severe the impact, the higher the possible anxiety and depression. The people with chronic diseases suffered higher possibilities of anxiety and depression, and the percentages of possible anxiety and depression increased with having more chronic diseases. The participants with moderate to bad self-rated health had a higher prevalence of anxiety (*OR* = 1.88, 95% *CI*: 1.53–2.31) and depression (*OR* = 1.37, 95% *CI*: 1.65–2.48) than persons with good health. The participants who reported severe perceived risk of COVID-19 infection were more vulnerable to anxiety (*OR* = 1.75, 95% *CI*: 1.42–2.17) and depression (*OR* = 1.37, 95% *CI*: 1.11–1.69) than people who reported low to moderate risk levels. The people aged 18–39 years were more prone to anxiety (*OR* = 1.45, 95% *CI*: 1.13–1.85) and depression (*OR* = 1.67, 95% *CI*: 1.32–2.12) than people aged 40–59 years.

**Table 4:** Multivariate logistic analysis of anxiety and depression of the adult, working-age population in Mainland China in the COVID-19 pandemic.

Dependent variables	Independent variables	Reference	*B*	SE	Wald	*OR*	95%*CI*	*p*
Anxiety	Big (very much to an extreme amount) impact on medical service needs	None	1.62	0.19	72.75	5.07	3.49–7.36	<0.01
	Small (a little to moderate) impact on medical service needs		0.68	0.15	21.12	1.97	1.48–2.64	<0.01
	Moderate to bad self-rated health	Good	0.63	0.10	36.15	1.88	1.53–2.31	<0.01
	2 or more chronic diseases	None	0.81	0.17	22.19	2.25	1.60–3.15	<0.01
	1 kind of chronic diseases		0.40	0.14	8.48	1.49	1.14–1.94	<0.01
	Severe perceived risk of COVID-19 infection	Low to moderate risk levels	0.56	0.11	26.72	1.75	1.42–2.17	<0.01
	No child	One or more children	0.46	0.12	15.01	1.58	1.25–1.99	<0.01
	Age (years): 18–39	40–59	0.37	0.13	8.56	1.45	1.13–1.85	<0.01
	Unemployed	Employed	0.35	0.15	5.35	1.42	1.05–1.90	0.02
	Junior or regular college educational background	Other	0.25	0.10	5.60	1.28	1.04–1.57	0.02
Depression	Big (very much to an extreme amount) impact on medical service needs	None	1.43	0.19	58.65	4.18	2.90–6.02	<0.01
	Moderate impact on medical service needs		0.84	0.17	23.98	2.31	1.65–3.24	<0.01
	A little impact on medical services		0.61	0.15	17.27	1.84	1.38–2.46	<0.01
	Two or more chronic diseases	None	1.03	0.18	33.53	2.80	1.98–3.97	<0.01
	One kind of chronic diseases		0.47	0.14	12.18	1.61	1.23–2.10	<0.01
	Moderate to bad self-rated health	Good	0.70	0.10	45.50	2.02	1.65–2.48	<0.01
	Age (years): 18–39	40–59	0.51	0.12	17.50	1.67	1.31–2.12	<0.01
	Unmarried^a^	Married	0.37	0.11	10.85	1.45	1.16–1.81	<0.01
	Severe perceived risk of COVID-19 infection	Low to moderate risk levels	0.31	0.11	8.33	1.37	1.11–1.69	<0.01

*SE, standard error; OR, odds ratio; and CI, confidence interval*.

a *Including divorced or widowed; Anxiety: Nagelkerke *R*^2^ = 0.177; Depression: Nagelkerke *R*^2^ = 0.163*.

Finally, participants with no children, with a junior or regular college educational background, or those who were unemployed had a higher prevalence of anxiety than the people with ≥1 child, with other educational backgrounds, or who were employed (Table 4). The unmarried (including divorced or widowed) had a higher risk of depression than married adults (*OR* = 1.45, 95% *CI*: 1.16–1.81).

## Discussion

This study describes anxiety, depression, and their associated factors among the working-age, adult population in Mainland China. It fills in the knowledge gap for this population group and suggests the need for targeted countermeasures to mitigate the negative psychological effects the pandemic has had on this focused population.

We found that 44.5% (17.6%) and 49.2% (21.3%) of the sample population suffered anxiety and depression (moderate-to-severe anxiety and depression) at the early remission stage of the COVID-19 pandemic, respectively. A survey conducted in a similar period in Shandong province, China, using GAD-7 and PHQ-9, reported that 20.8% and 19.5% of the general public had moderate-to-severe anxiety and depression symptoms, respectively (Zhang et al., 2020a). Another study among young adult university students during the same pandemic stage as our study in China revealed that 34.73% had anxiety symptoms, and 46.55% reported depressive symptoms (Sun et al., 2021). All of these results indicate that the general adult population in China might suffer similar levels of anxiety and depression in the early regular control period of the pandemic. In 2012, prior to the COVID-19 outbreak, the weighted prevalence of anxiety and depression of adult residents was 5.0% and 3.6%, as reported by the China mental health survey (Huang et al., 2019), respectively. By using the same measurement tools that were used in the pre-pandemic study, this study highlights the mental health impact of the pandemic. The prevalence of moderate-to-severe anxiety among a national sample of 38,294 Chinese urban adult dwellers was 5.3% (Yu et al., 2018), and the prevalence of depression among adult participants was 6.2% and 33.6% in Jiangsu province and in Chaoyang district, Beijing, respectively (Zhu et al., 2013; Tu et al., 2018). This suggests that the levels of anxiety and depression of our participants had all increased remarkably compared to before the pandemic in Mainland China. After literature review focused on the peak of the COVID-19 pandemic (from the end of January 2020, to February 2020) in Mainland China, we found the prevalence of anxiety and depression (moderate-to-severe anxiety and depression) of the general adult population was 44.6% (16.8%) and 53.5% (24.1%; Liu et al., 2020b), respectively, while working-age residents in Wuhan reported 55.9% (27.9%) and 57.4% (25.2%) anxiety and depression (moderate-to-severe anxiety and depression), respectively (Yang et al., 2020). In a word, compared with the prevalence of anxiety and depression at the peak of the pandemic in Mainland China, the anxiety level of the participants had remain almost unchanged, while the depression level declined slightly during the stage of regular control of COVID-19. For a broader perspective on these results, a systematic review across 33 countries (*N* = 640,037) discovered that the overall proportion of study participants with moderate-to-severe depression, for example, was 21.4% (Lee et al., 2021). Another study reported that 49.5% (19.2%) of the Saudi residents aged 16 years and above suffered anxiety symptoms (moderate to severe anxiety; Alqahtani et al., 2021). The findings of the above two studies show that our participants in China had similar mental health status as comparable populations in some other counties. But we also noticed that in some cases there were different results at home and abroad. Wang et al. reported that remission of the pandemic was associated with greater prevalence of severe depression and anxiety among adults living in the United States (Wang et al., 2021f), with the mean (SD) of the GAD-7 being 6.56 (5.36; Wierenga et al., 2021), which was higher than that of our participants.

This study revealed that over four fifths (83.7%) of the participants Mainland China reported a negative impact on their medical service needs, and the percentages of possible anxiety and depression among people who reported a big impact (15.4%) were 5.07 times and 4.18 times those of people who reported no impact. The possibilities of anxiety and depression among people who reported a little to moderate impact were 0.84–1.31-fold higher than those of persons who claimed no negative impact. Meanwhile, adults with moderate-to-bad health or chronic diseases suffered higher percentages of anxiety and depression than those with good health (Wang et al., 2021b) or without chronic diseases (Wang et al., 2021d), with more chronic diseases predicting higher possibilities of anxiety and depression. The study of Wang et al. has reported that a history of chronic diseases resulted in a higher risk of total psychological outcomes, including moderate-to-severe symptoms of depression and anxiety (Wang et al., 2021c). The people with more chronic diseases or lower health levels probably need to visit doctors more frequently than healthier people, however, their routine or unexpected medical service needs were inevitably and negatively affected due to pandemic control policies and measures in medical institutions (Wang et al., 2020a), movement restrictions of populations, and city neighbourhood/rural village access management during the pandemic period, in which case the negative impact would in turn lead to an increased risk of anxiety and depression. Zhang et al. identified that 88.1% of participants with chronic diseases were afraid of being infected with COVID-19 while seeing a doctor in the hospital (Zhang et al., 2020b); a previous study revealed that 59.8% of the western medical treatment of breast cancer patients was delayed or interrupted for 4 weeks or more in Mainland China (Chen et al., 2020). Nevertheless, another survey conducted in British Columbia, Canada, found that having a chronic illness was not associated with greater anxiety or depression symptoms during the COVID-19 pandemic (Budu et al., 2021), which might be correlated with that country’s public health system, the hierarchical nature of that health service system, and the initial peak period of the pandemic in that province. Overall, it could be inferred that the negative impact on medical service needs was the principal risk factor for anxiety and depression during the normal COVID-19 control stage, suggesting that in future pandemics the necessity and urgency of maintaining medical service capacity while in the shadow of the public health emergency will be crucial. To meet this need, online medical services and telemedicine might be important options, among other feasible countermeasures (Shah et al., 2020; Wang et al., 2020b; Graziano et al., 2021). Furthermore, research on how to restore service capacity as soon as possible to meet the need for routine and delayed medical treatment in the post-pandemic period should be a part of the priority agenda.

Coronavirus 2019 is a new coronavirus showing a high risk of human-to-human transmission (Rothe et al., 2020). Limited knowledge about COVID-19 may cause anxiety, especially among individuals in severe outbreak regions. This study showed that the severe perceived risk of COVID-19 infection predicted higher possibilities of anxiety and depression. Guo et al. reported that the number of confirmed cases in a respondent’s city of residence were positively associated with symptoms of depression (Guo et al., 2021). Zhen and Zhou found that COVID-19 infection-related personal experience and risk perception could predict higher odds of anxiety (Zhen and Zhou, 2020), while Wu et al. discovered that physical distance to confirmed cases had a significant influence on citizens’ anxiety (Wu et al., 2021a). Considering the fact that the pandemic is under control in Mainland China as of March 2020 and social life is by and large restored, the psychological health level of the population should be expected to increase in 2021. A survey conducted in the Chinese general adult population from February 2 to 9, 2021, showed this trend preliminarily, though the proportion of people with depression and anxiety symptoms was still high even 1 year after the peak of the COVID-19 epidemic in China (Qi et al., 2021). At the height of the pandemic, the participants in Wuhan had a significantly higher prevalence of anxiety, depression, and other mental health problems than the people in other areas in Mainland China (Wang et al., 2021a). Though the participants in Wuhan reported the highest percentage of severe perceived risk of COVID-19 infection in the remission stage of the pandemic, our results did not demonstrate that they were more susceptible to anxiety and depression, which suggests that the province/city was not a factor associated with anxiety or depression any longer. The stringent measures implemented by governments to control the spread of COVID-19 benefited not only the physical, but also the mental, health of the population.

We found that adult people younger than 40 years were more prone to anxiety and depression than the older group, a result that was consistent with a study in Saudi residents (El Keshky et al., 2021), from which it could be inferred that the mental health of younger and middle-aged people deserved special consideration in the normal pandemic control stage. Our results indicated that marriage was a protective factor against depression (Wang et al., 2021b), which might be attributed to the mutual emotional and economic support to be found in the family, especially given the background of a significant decrease of social activities. According to our analysis, however, marriage was not correlated with the anxiety of the participants in the regular control period of the pandemic. Distinct from our results, Wang et al. (Wang et al., 2021b) reported that married residents in Hubei had a lower risk of suffering anxiety at the peak of the pandemic, and the non-married participants in quarantine in Shenzhen showed higher risks of depression and anxiety symptoms from April to June, 2020 (Wang et al., 2021d).

Compared to those with no children, participants with one or more children had less anxiety, but whether they had children was not an associated factor for depression in this study. A study conducted in Asia reported that staying with children was associated with lower stress, anxiety, and depression (Wang et al., 2021e), and having one child was a protective factor in healthcare workers’ psychological distress (Zhang X. et al., 2021). The possible reason might be that taking care of children may require a personal sense of duty and provide a sense of achievement, so as to provide a certain psychological buffer during the long-term stay-at-home period. Employed participants showed better mental wellbeing in the dimension of anxiety based on our results, as Solomou and Constantinidou have also reported (Solomou and Constantinidou, 2020), which may be related to the sense of security that having a steady personal income can provide, though personal income was not an associated factor in our study.

Ni et al. (2021) discovered that participants with a college-level educational background had a higher rate of anxiety and depression, although the latter had no statistical significance. Generally consistent with our study, and revealed that having completed college was an independent correlate of having anxiety symptoms (Ni et al., 2021). With regard to there being more anxiety in participants with a college-level educational background than that those with a lower educational level, Fu et al. (2020) and Wang et al. (2021e) also found that participants with higher education had higher anxiety and suggested possible reasons: First, compared with a lower educational level, participants with a college-level educational background might have higher eHealth literacy (Tennant et al., 2015), which sparked their anxiety levels if they spent increased time searching for and reading information on COVID-19 (Guo et al., 2021); second, the proportion of urban participants among those with a lower educational level was significantly lower than that of participants with a college background (*p* < 0.01), however, urban residents had higher levels of anxiety than rural residents in China during the pandemic (Zhang J. et al., 2021), so the increased levels of anxiety may be related to location rather than education. Participants with a graduate educational level had less anxiety than those with a college educational background, according to a study conducted in Cyprus which found that bachelor-level students had higher anxiety and depression scores compared to PhD students during the pandemic (Solomou and Constantinidou, 2020). The possible reason might be that people with graduate backgrounds have more concrete personal goals and know how to make good use of their time during long-term stay-at-home periods than those with a lower level of college education, so they showed better psychological health. The specific reasons why people with low levels of education and with graduate backgrounds had less anxiety than people with college-level education merits further research. Finally, sex was not a significant factor for anxiety and depression in our study, though a previous study showed that rural female respondents had higher mental health risks during the pandemic (Jia et al., 2021), and other research found that males in the general population had higher odds of poor psychological outcomes than females (Wang et al., 2021c).

## Limitations

This study has a few limitations to acknowledge. First, a non-probabilistic snowball or convenience sampling was used in this study, hence, the informativeness of the findings reported here is limited by the disproportionally representative nature of the population samples and by this potential selection bias, so we must be prudent when generalizing the results to other regions of China. For example, 79.1% of our participants have associates degrees or higher. People with no college educational background might have had little interest in participating in the survey and be underrepresented. Thus, we could recruit only a few respondents with lower education backgrounds. Second, some important data was not collected, such as related information-seeking variables (Pan et al., 2020). Liu et al. (2021) found that respondents who spent considerable time browsing excessive negative information related to the pandemic displayed an increased likelihood of anxiety during the pandemic, and Tan et al. (2021) reported that COVID-19 induces long-term negative effects on online public sentiments even as the country recovers from the pandemic. Finally, this study was cross-sectional, and a follow-up study should be implemented to trace the changing pattern of mental health in adult, working-age people in Mainland China given the background of the COVID-19 resurgence induced by the highly infectious Delta variant and other virus variants imported from abroad, as well as in the post-COVID-19 period.

## Conclusion

In summary, the anxiety and depression of our participants were still at a relatively high level during the normal pandemic control period. The impact of the pandemic on meeting medical service needs was the first-rank predictor of anxiety and depression, followed by self-rated health status and chronic diseases. Adults of a younger age or those who had a severe perceived risk of COVID-19 infection showed higher risks of anxiety and depression symptoms. Having no children, unemployment, and a college educational background were associated with higher anxiety levels, whereas participants who were single had higher odds of depression. To meet the health service needs of this group and to mitigate the risk of depression and anxiety symptoms in times of crisis, the most important suggested solutions were telehealth and full implementation of a hierarchical medical system in the context of limited high-quality service resources in Mainland China.

## Data Availability Statement

The original contributions presented in the study are included in the article/supplementary material, further inquiries can be directed to the corresponding authors.

## Ethics Statement

The studies involving human participants were reviewed and approved by the ethics committee of the School of Public Health at Fudan University in Shanghai, China. The patients/participants provided their written informed consent to participate in this study.

## Author Contributions

FC and HX conceived the idea. XH, SW, and HX substantially contributed to the design of the study and to the acquisition of data. FC performed data cleaning and drafted the manuscript. QZ revised the manuscript. YW and XZ participated in the statistical analysis and interpretation of the results. All authors contributed to the article and approved the submitted version.

## Funding

This work was supported by the National Natural Science Foundation of China (71673052), a research project in the humanities and social sciences of Anhui Colleges and Universities (SK2019A0182), the Songjiang Scientific and Technological Project in 2020 (20SJKJGG242), the 111 Project (B16031) from the Department of Education (US), and as a major project of the National Social Science (no. 17ZDA078) from the National Office for Philosophy and Social Sciences (CN).

## Conflict of Interest

The authors declare that the research was conducted in the absence of any commercial or financial relationships that could be construed as a potential conflict of interest.

## Publisher’s Note

All claims expressed in this article are solely those of the authors and do not necessarily represent those of their affiliated organizations, or those of the publisher, the editors and the reviewers. Any product that may be evaluated in this article, or claim that may be made by its manufacturer, is not guaranteed or endorsed by the publisher.
